# Endothelial hypoxic metabolism in carcinogenesis and dissemination : HIF-A isoforms are a NO metastatic phenomenon

**DOI:** 10.18632/oncotarget.1461

**Published:** 2013-11-18

**Authors:** Cristina Branco-Price, Colin E. Evans, Randall S. Johnson

**Affiliations:** ^1^ Department of Physiology, Development and Neuroscience, University of Cambridge, Cambridge, UK

**Keywords:** Vascular endothelium, hypoxia, metastasis, HIF, HIF isoforms, nitric oxide, cell-specific responses

## Abstract

Tumor biology is a broad and encompassing field of research, particularly given recent demonstrations of the multicellular nature of solid tumors, which have led to studies of molecular and metabolic intercellular interactions that regulate cancer progression. Hypoxia is a broad stimulus that results in activation of hypoxia inducible factors (HIFs). Downstream HIF targets include angiogenic factors (e.g. vascular endothelial growth factor, VEGF) and highly reactive molecules (e.g. nitric oxide, NO) that act as cell-specific switches with unique spatial and temporal effects on cancer progression. The effect of cell-specific responses to hypoxia on tumour progression and spread, as well as potential therapeutic strategies to target metastatic disease, are currently under active investigation. Vascular endothelial remodelling events at tumour and metastatic sites are responsive to hypoxia, HIF activation, and NO signalling. Here, we describe the interactions between endothelial HIF and NO during tumor growth and spread, and outline the effects of endothelial HIF/NO signalling on cancer progression. In doing so, we attempt to identify areas of metastasis research that require attention, in order to ultimately facilitate the development of novel treatments that reduce or prevent tumour dissemination.

## INTRODUCTION

In the last decade, tumors have been redefined as multicellular organs, a notion that replaced the previously confined view that tumors are pluricellular masses [1], and which restricted both the understanding of the biology of cancer, and the development of effective therapeutic strategies. Specific and substantial roles of non-malignant cells in cancer progression (tumor growth and metastasis) have recently been elucidated, and therapeutic focus has begun to shift away from the tumor cells themselves towards a broader spectrum of cellular and molecular targets [2, 3], all inter-dependent components of a plastic, adaptive and mobile organ with powerful survival strategies. Despite vast research on the tumor microenvironment, however, alternative cancer treatments aimed at non-malignant cells remain elusive [4-6].

The processes of tumor growth and dispersion are highly complex, given that each cell type responds to different stimuli in a unique fashion. Multiple biochemical, mechanical, and signalling pathways define disease advancement, and in doing so, modulate clinical outcome [5-7]. Assessing the progression of cancer is currently forced to incorporate this organic complexity whereby the contribution of different cell types to the processes of tumor growth, aggressiveness, dispersion and response to therapy is accounted for. Therefore, physiological and metabolic adaptations of cells within the tumor and metastatic environment have to be assessed individually and taking into consideration the synergistic impact on the others [7, 8].

**Figure 1:**
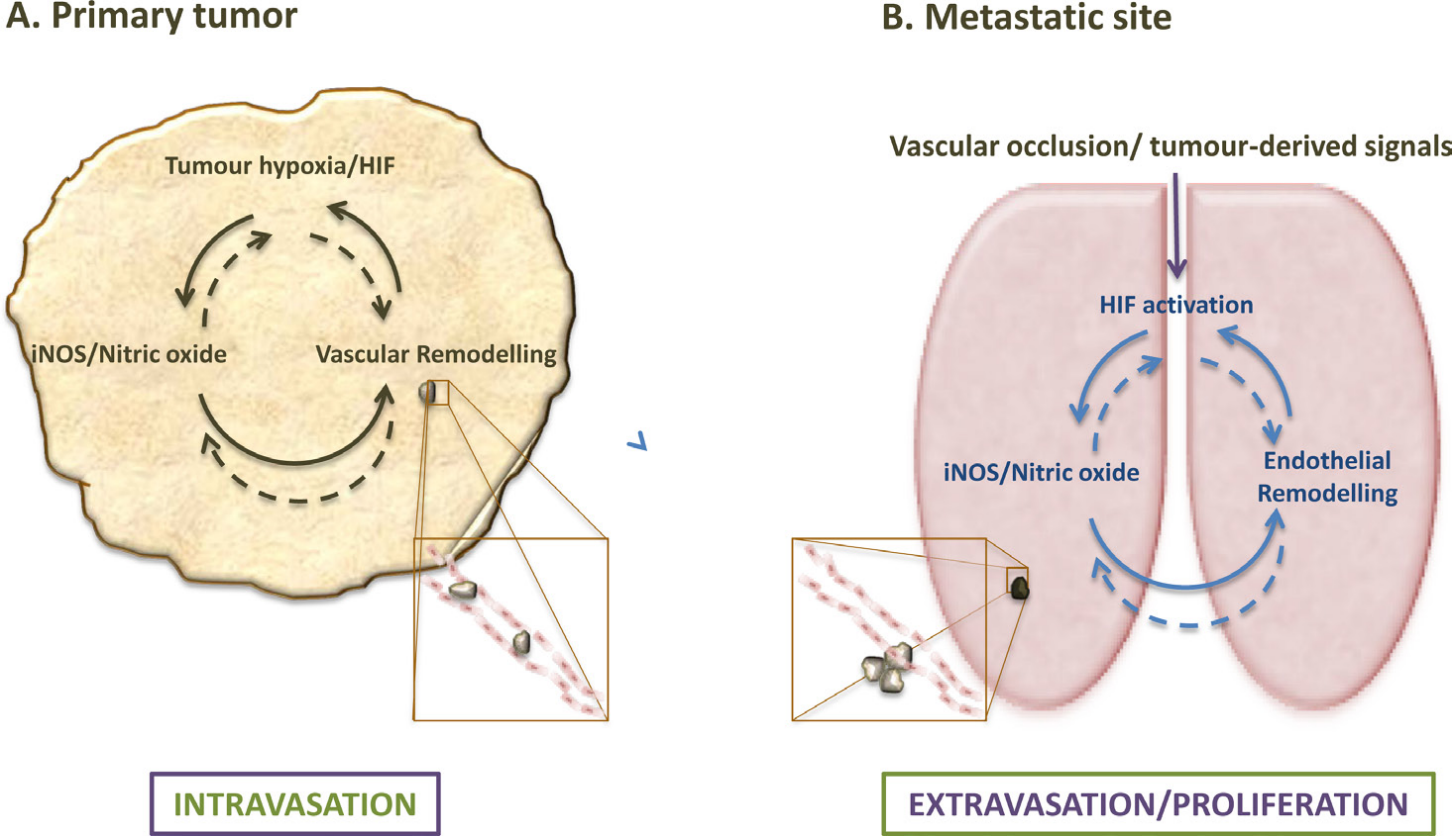
Endothelial HIF/NO-mediated regulation of metastases at primary tumor and metastatic sites (A) Hypoxia and subsequent HIF-a stabilization are common trademarks of solid tumors, which affect the myriad of cellular components within the tumor, and lead to cell type-specific responses, including NO-mediated endothelial remodelling that increases vascular leakiness and drives intravasation. (B) Endothelial HIF and NO signalling also affect extravasation and possibly post-extravasation proliferation of tumor cells; remodelling responses to HIF activation and NO induction in the endothelium, however, can be tissue- and isotype-specific.

Tumor growth and spread is strongly affected by, for example, the endothelial cell (EC) response to hypoxia [9]. We and others recently demonstrated that this response controls metastatic success, to a significant extent, via hypoxia-inducible factors (HIFs) [10, 11] and nitric oxide (NO) [11]. Our study unveiled a role for endothelial HIF/NO-dependent signalling in metastasis, which is differentially regulated by the two major HIF isoforms (HIF1-alpha and HIF2-alpha) that severely impact upon the ability of tumor cells to successfully establish distant metastases [11]. In this study, HIF1-alpha was shown to have a pro-metastatic effect, while HIF2-alpha opposed metastasis, and although we speculated that these effects occur via NO- and vascular endothelial growth factor (VEGF)-mediated changes in vessel permeability (Figure 1), the mechanisms by which endothelial HIF/NO signalling intervenes in metastasis are not fully understood. The aim of this perspective is to summarise the diverse functions of endothelial and HIF/NO signalling during cancer progression, and to discuss the potential impact of these findings on future studies leading towards the development of novel adjunctive cancer therapies.

### HIF-mediated response to hypoxia during cancer progression

Hypoxia is a trademark of solid tumors that is associated with poor prognosis and successful metastases [12-14]. The HIF-mediated control of this response has been comprehensively characterized [15-18], and the role of these transcription factors is presently acknowledged to be cell-specific [19]; mechanisms and factors regulating HIF-a stability as well as the target genes and pathways regulated by them vary between different cell types and in different conditions, which include stimuli other than hypoxia [12, 19].

For that reason, the attractive strategy of targeting HIF1-alpha for cancer therapy needs to be observed and integrated in a multicellular and temporal context; Although HIF1-alpha (and in some cases HIF2-alpha) activity in tumor cells, for example, is a clear indicator of poor prognosis [12, 17, 20], in other cell types this may not be the case [11]; many other non-malignant cell types within the tumor microenvironment have been shown to significantly contribute to the regulation of cancer progression [3], especially macrophages [21, 22] and fibroblasts [23] which, among other things, greatly contribute to the paracrine VEGF signalling that leads to aberrant vascularization. ECs, specifically, both at the tumor stroma [24] and at the extravasation sites [11] also have unique responses to hypoxia [25, 26] and play a critical role tumor dispersion [24].

### Endothelial cell-specific response to hypoxia

ECs are mostly glycolytic and their hypoxic response is triggered at lower oxygen concentrations compared with other cell types [25]. Although the machinery and capacity to respond to less severe hypoxia is present and functional in ECs, this discrepancy appears to involve intricate mechanisms, and the activation of HIF in these cells is sensitive to minor oscillations in oxygen tension as well as other chemical signals [26-28].

Reductions in oxygenation that occur in a solid tumor directly impact upon the endothelium, which can act as a barrier or vehicle for metastasising cells. Hypoxic conditions, as mentioned above, are associated with a leaky and inefficient vasculature that compromises the barrier function of the blood vessels against infiltrating cells. At the site of extravasation, hypoxia could occur locally, for example, when migrating cells arrest in the capillary bed prior to seeding at secondary sites [29, 30].

In the case of the characteristic tortuous and inefficient tumor vasculature, the blood flow and consequently oxygen delivery is insufficient. ECs sense and respond to this stimulus by activating mechanisms that lead to increased proliferation; this is supported by an increase in endogenous and exogenous VEGF levels and associated receptors, migration (required for vascular branching and angiogenesis) and activation of hypoxic metabolism that ensures survival and amelioration of the hypoxic condition [31]. Specifically, tumor ECs: (i) enable the delivery of oxygen and nutrients to the tumor; (ii) form a physical barrier between the tumor mass and the remaining organism; (iii) act as a vehicle through which detached tumor cells travel to distant organs; and (iv) provide an anchor required for survival of micrometastases [32, 33]. By directly interacting with other cell types in the tumor microenvironment (*e.g.* inflammatory cells, tumor cells, platelets), ECs can aid or impair tumor growth and spread [7, 34, 35].

Mechanisms that regulate the generation and function of the tumor vasculature have therefore been the target of many experimental and clinical studies that aim to reduce tumor propagation [36]. Although tumor size is often initially reduced by anti-angiogenic treatments (*e.g.* HIF and VEGF inhibitors), such therapies frequently have short-lived effectiveness, and long term benefits are not only seldom observed, but can also result in higher metastatic incidence [4, 37]. These findings have highlighted the complex and multi-factorial nature of the HIF-mediated angiogenic response to hypoxia during cancer progression [27, 38], one component of which is regulated solely by EC-specific HIF.

### HIF-mediated response in hypoxic EC during cancer progression

Effects of EC-specific HIF signalling on cancer progression have been elucidated in the last decade, often using Cre-LoxP-mediated deletion of EC HIF-alpha driven by the Tie2 or VE-cadherin promoters in mice [11, 12]. Mice with EC-specific deletion of HIF1-alpha, for example, develop smaller subcutaneous tumors compared with those implanted into wild-type littermates [27]; EC-specific deletion of HIF2-alpha compromises neovascularization and vessel remodelling [38]. EC-specific HIF1-alpha knockout also reduces metastases in the Polyoma middle T (PyMT) mouse model of mammary cancer, and attenuates extravasation in mice subjected to intravenous tumor cell injections [11].

Studies using mouse models of cancer with EC-specific deletion of HIF2-alpha have also shown reduced tumor growth rates compared with wild type mice [10], but the effect of EC-specific HIF2-alpha deletion on metastatic efficiency in these mice has been reported as being advantageous [10] and deleterious [11]. The role of the endothelium in mediating metastatic disease, the location and timing of HIF-signalling and the contribution of NO to cancer progession is a puzzle with possibly multiple solutions. It is therefore worthwhile identifying the pathways activated downstream of these main regulators as potential targets for therapy, in alternative or in addition to targeting them directly.

The role of EC-specific HIF signalling in metastatic disease is an active area of research in our laboratory. In multiple cell types, one important downstream effector of the HIF-mediated responses is the metabolic regulator, NO [39-41]. In addition to all the functions performed by this highly reactive molecule, there is currently a plethora of information that directly correlates NO with cancer progression, metastases and clinical outcome [42, 43].

### Response to hypoxia during cancer progression: contribution of Nitric Oxide

NO is an ephemeral but highly reactive molecule with direct cytotoxic effects [44]. NO can be generated as a result of hypoxia, given that the inducible form of nitric oxide synthase (iNOS) is a target of HIF1-alpha [45, 46]. Conversely, NO can also be a cause for metabolic hypoxia, given that competition with oxygen at cytochrome *c* results in diversion of oxygen to other pathways [46, 47]. NO has also been shown to increase HIF1-alpha degradation during hypoxia [48], and is negatively correlated with blood oxygen saturation, for example during arthritis development [49].

### NO signalling in cancer progression and metastases

During carcinogenesis, NO is generated by different cell types, in different amounts, and via different regulatory mechanisms [39]. In human breast tumors, for example, higher levels of NO are found in malignant tissues compared with benign masses, and these levels increase as tumors progress, independent of other prognostic parameters such as estrogen receptor level, human epidermal growth factor receptor (HER) or menopausal status [50].

Although the role of NO is well established in other pathological settings, such as the regulation of vascular tone, the inflammatory response, and mitochondrial respiration [48, 51-53] its impact on tumor progression and metastatic processes appears ambiguous [44, 54]. Studies have previously suggested both facilitating and hampering effects of NO on tumor growth and metastatic success.

NO is known to function as a pro-apoptotic agent [54-56] and as such can promote tumor cell death [39]. This property has lead to the suggestion that NO donors could be used to limit tumor growth, and it has also been argued that NO donors could improve efficacy of chemo and radiotherapy by counteracting desensitization and resistance [57-59].

On the other hand, findings that argue for a pro-tumorigenic role of NO are varied. For example, excessive levels of NO and subsequent oxidative stress, lead to DNA and protein damage, thereby increasing mutation rates and consequently enhancing tumor aggressiveness [44, 55]; Furthermore, iNOS expression in many human and murine cancers correlates with poor prognosis and decreased disease-free survival [41, 60]. NO is also known as a mediator of angiogenesis [41], therefore contributing to tumor growth and dissemination. NO-driven metabolic hypoxia was also shown to affect angiogenesis, hyperplasia, and the formation of inflammatory lesions breast cancer patients [50] while iNOS depletion or inhibition in murine breast cancer models consistently results in reduced tumor growth and metastases [11, 61, 62]. The presence of NO has also been shown to decrease immune cell-mediated tumor cell death via inhibition of HIF1 under hypoxia [59], and chemokine nitration within the tumor microenvironment appears to be one of the mechanisms by which tumor NO prevents T-cell infiltration [40, 63].

Although NO is clearly produced in tumor cells [40, 41, 60, 61, 64], alternative cellular sources of NO are seldom reported, as are their spatial and temporal expression patterns of the NOS isoforms. Moreover, metabolic effects of NO on cancer progression at the primary tumor site and particularly at the site of metastasis require further investigation.

### HIF and/or NO: coordinated regulation of EC metabolism during tumor growth and metastasis

A number of studies have investigated the potential effects of endothelial HIF-mediated NO signalling on cancer progression (Table I). It has been shown, for example, that cyclic hypoxia (likely induced by heterogeneous and deregulated blood flow in solid tumors) increases EC survival through HIF1-alpha stabilization, and that eNOS attenuates HIF1-alpha expression and interferes with cell respiration [26, 65]. Furthermore, EC survival in response to cyclic hypoxia is increased by NOS inhibition [65].

**Table 1:** Studies investigating the role of endothelial HIF/NO/VEGF signalling in cancer progression

Methods	Results	Conclusions	Ref
EC HIF1-alpha and 2-alpha knockout mice with: Implanted subcutaneous and spontaneous mammary tumors Intravenous Lewis lung cancer cells Migration of cancer cells across ECs	EC HIF1-alpha knockout reduced: NO synthesis Cancer cell migration across ECs Metastatic success EC HIF2-alpha knockout had the opposite effect	EC HIF1-alpha and HIF2-alpha have opposing roles in the regulation of metastatic seeding HIF-alpha acts in a cell- and isoform-specific manner	[11]
Analysis of human mammary tumor	eNOS, HIF1-alpha, iNOS, and VEGF, were present There were correlations between: eNOS and HIF1-alpha, VEGF, or VEGFR2 VEGF and VEGFR2	HIF1-alpha/NO/VEGF signalling appears to be active in human breast cancer	[87]
EC HIF2-alpha knockout mice with autochthonous skin tumors Migration, proliferation, adhesion, and invasion of HIF2-alpha knockout ECs	EC HIF2-alpha knockout impaired: Functional tumor angiogenesis tumor perfusion Adhesion to collagen EC HIF2alpha knockout increased: Migration and invasion Tube formation	EC HIF2-alpha is required for tumor vessel growth Roles of EC HIF1alpha and 2alpha in pathological angiogenesis are distinct	[38]
Analysis of human esophageal tumor	HIF1-alpha was present in tumors There were associations between: HIF1-alpha, VEGF, and iNOS eNOS and VEGF	HIF1-alpha, iNOS, and VEGF expression is linked in human esophageal cancer	[88]
EC HIF2-alpha knockout mice with xenograft tumors Vascularisation, apoptosis, and adhesion of HIF2alpha knockout ECs	EC HIF2-alpha knockout impaired: Vessel integrity EC structure tumor angiogenesis EC adhesion	EC HIF2-alpha is required for normal EC function and vessel formation EC HIF1-alpha and 2-alpha have distinct roles in angiogenesis	[10]
EC HIF1-alpha knockout mice with implanted subcutaneous tumors Proliferation, migration, invasion, and tube formation of HIF1-alpha knockout ECs	EC HIF1alpha knockout inhibited: Proliferation, migration, and invasion tumor angiogenesis and growth	EC HIF1/VEGF signalling is crucial for cell function during tumor angiogenesis	[27]

NO mimetic agents have also been shown to suppress HIF1-alpha accumulation under hypoxia [66]; interestingly, several studies report that the accumulation of angiogenic VEGF is directly correlated with NO levels [67, 68]. We showed that EC-specific VEGF levels decrease in the absence of iNOS expression or activity and increase in the presence of an NO donor, and that this occurs in direct correlation with metastatic success [11]. iNOS-dependent VEGF production has also been reported elsewhere [64, 69, 70].

The EC response to hypoxia affects processes that contribute to tumor progression and spread, including vessel permeability, tumor cell migration, proliferation, and interaction with other cells relevant for tumor cell seeding and establishment, including inflammatory cells and platelets [71-73] NO stimulates proliferation, migration, and differentiation of ECs to form new blood vessels [28], but again positive [74] and negative [75] effects of NO on vascular permeability have been reported.

Contrasting effects of endothelial HIF/NO signalling on cancer progression could again be a result of differences in cell/tissue type, NOS isoform, and NO levels between studies. Most studies on endothelial-derived NO production have been circumscribed to the levels and activity of eNOS, which is constitutively expressed and widely accepted as the major determinant of NO production in ECs. It has also been reported, however, that the major function of eNOS is to maintain residual levels of NO, which in turn ensures that endothelial permeability and vascular tension are stable [45]. Conversely, sharp and dramatic increases in NO levels are usually a result of iNOS activit; iNOS is more abundant and readily induced in murine compared with human ECs, which are more reliant on concomitant cytokine stimulation in addition to HIF1-alpha activation [45]. Endothelial cells from different tissues and organs, whether in physiological or cultured conditions also display different gene expression and stress responses [76, 77]; this is yet another level of intricacy of the mechanisms underlying cell-cell interactions that control or predispose certain vascular beds to metastatic events.

Given that EC behaviour varies between organs [77, 78], the effect of EC HIF/NO signalling on tumor growth and metastasis may also depend on cancer type and metastatic site. NO production in mouse models of breast cancer is directly correlated with VEGF levels, but the pathway(s) that link cell-specific HIF/NO signalling with cancer outcomes such as tumor growth and metastasis remain incompletely understood. One current aim in our laboratory is therefore to fully characterise the HIF/NO-mediated response to hypoxia in ECs, and to study downstream effects of EC HIF/NO signalling on cancer progression, using mouse models of cancer and cell-specific deletion of HIF-alpha or iNOS.

## PERSPECTIVES: CURRENT AND FUTURE WORK

**Figure 2:**
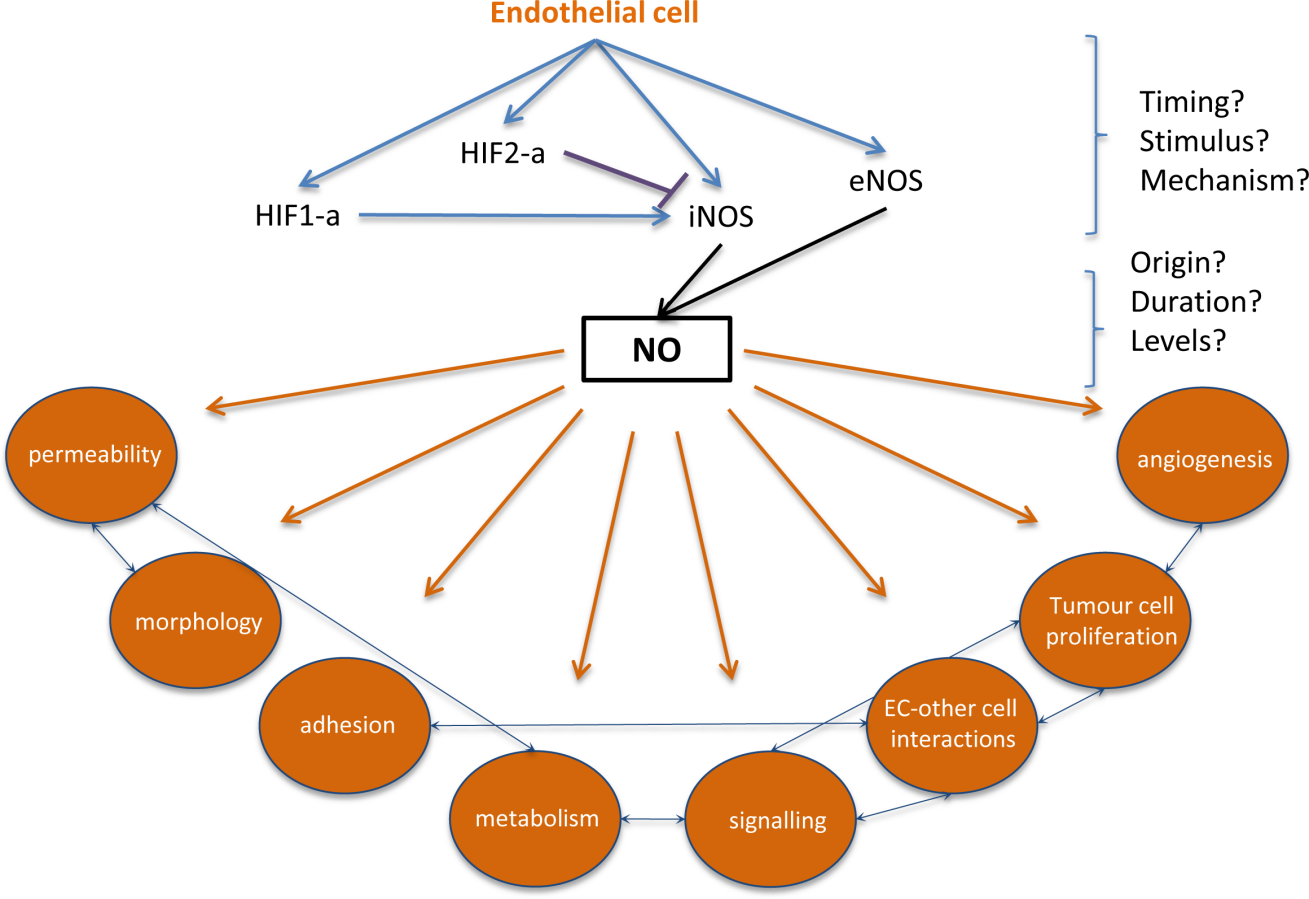
Filling the gaps in endothelial HIF-alpha, NO signalling and subsequent changes that can contribute to metastatic events At the site of extravasation, endothelial HIF-1a or HIF-2a activation results in differential activation of iNOS, with direct influences on NO production. Hypoxia- and HIF-independent signals can also stimulate NO production, and the origin, concentration, and duration of NO may affect metastatic success. Downstream effects of endothelial HIF/NO signaling, such as endothelial permeability and endothelial cell-tumor cell adhesion, could be targeted to reduce or prevent extravasation.

Key questions remain when characterizing the role of the vasculature in metastatic disease, *e.g.* what roles do EC-specific HIF and NO play during intravasation, and what cell-specific molecular signalling pathways regulate the extravasation and viability of colonizing cancer cells? Although mechanisms that regulate endothelial HIF/NO-mediated cancer progression are not fully understood, therapeutic or gene-mediated disruption of this signalling cascade could potentially reduce metastatic success (*Figure 2*).

In the primary tumor microenvironment, it is known that hypoxia triggers HIF1-alpha stabilization in different cell types. Subsequently, target genes including VEGF are transcriptionally up-regulated and cooperate to facilitate tumor growth and intravasation [79]. Factors that regulate stabilization of the HIF isoforms and iNOS in ECs at the site of extravasation however, are largely unknown and may be hypoxia-independent [80, 81].

Transient hypoxic events are likely to occur when circulating tumor cells obstruct small capillaries, for example in the lungs. The molecular outcome of those events would depend on a myriad of factors including duration and severity of hypoxic stimulus, as well as cell type(s) affected, and this could ultimately determine the extent to which each HIF-alpha isoform is stabilised, and in turn affect local NO levels that control extravasation. HIF/NO signalling in ECs, for example, could regulate metastasis by affecting vessel permeability (via disruption of endothelial junctions) or interaction between the endothelium and tumor cells (via cell adhesion molecules). HIF and/or iNOS could also be stabilized at the site of extravasation prior to the arrival of colonizing tumor cells, as a result of tumor-derived signals that circulate systemically [82, 83] For example, hypoxia-independent chemical stimuli could arise from the primary tumor [15, 73, 83].

Given that cancer cell extravasation and metastatic colonization are rate-limiting steps that involve reciprocal interactions between tumor cells and host stroma [84-86], it will be crucial to understand the molecular and cellular mechanisms that regulate these interactions. To investigate whether endothelial HIF-alpha and/or iNOS have any role in assisting or preventing the establishment of tumor cells at secondary metastatic sites, lung endothelium can be exposed to hypoxia in EC-specific HIF-alpha null, NO null, or wild type mice (Branco-Price, unpublished data), and metastatic foci subsequently quantified using a mouse model of extravasation [11]. In order to characterise the spatial and temporal patterns of HIF-alpha isoforms and NO expression during cancer progression, we are also currently quantifying their levels at various metastatic sites and stages of tumor development.

Simple immunohistochemical and biochemical approaches can be used to investigate changes in cell-specific HIF-alpha isoform stabilization and their associated changes in vascular integrity that modulate metastasis. These techniques can also be used to localise hypoxic foci and predict the stage of tumor development at which HIF-directed therapy would be beneficial or detrimental. Discovering whether HIF activation is a result of local hypoxic loci and/or systemic signals from the primary tumor would improve our understanding of metastasis significantly, and elucidation of HIF/NO-mediated mechanisms that regulate tumor dispersion could lead to the development of novel therapies to counteract this process.

One of the questions that have to be addressed is which HIF-alpha isoforms in the lung endothelium of tumor bearing mice are active, alternatively or simultaneously, and what is regulating that equilibrium.

## CONCLUSIONS

The EC HIF/NO signaling pathway plays a crucial role in cancer progression. Such cellular and molecular pathways may be targeted in order to reduce tumor growth and spread. Given that: (i) hypoxic stress can cause differing levels of induction of the two HIF-alpha isoforms; (ii) HIF/NO-mediated responses to hypoxia during cancer progression are cell type-specific; and (iii) current HIF-alpha inhibitors are not cell type- or HIF isoform-specific, novel therapies should attempt to target individual HIF isoforms in defined cell sub-populations within the tumor microenvironment. Alternative or additional therapeutic approaches could also arise through targeting of factors downstream of EC HIF/NO.
